# The Role of Chemoattractant Receptors in Shaping the Tumor Microenvironment

**DOI:** 10.1155/2014/751392

**Published:** 2014-07-10

**Authors:** Jiamin Zhou, Yi Xiang, Teizo Yoshimura, Keqiang Chen, Wanghua Gong, Jian Huang, Ye Zhou, Xiaohong Yao, Xiuwu Bian, Ji Ming Wang

**Affiliations:** ^1^Laboratory of Molecular Immunoregulation, Cancer and Inflammation Program, Center for Cancer Research, National Cancer Institute, Frederick, MD 21702, USA; ^2^Endoscopic Center, Zhongshan Hospital, Fudan University, Shanghai 200032, China; ^3^Department of Pulmonary Medicine, Ruijin Hospital, Shanghai Jiaotong University School of Medicine, Shanghai 200025, China; ^4^Basic Research Program, Leidos Biomedical Research, Inc., Frederick, MD 21702, USA; ^5^Institute of Pathology and Southwest Cancer Center, Southwest Hospital, Third Military Medical University, Chongqing 400038, China; ^6^Department of Gastric Cancer and Soft Tissue Surgery, Fudan University Cancer Center, Shanghai 200032, China

## Abstract

Chemoattractant receptors are a family of seven transmembrane G protein coupled receptors (GPCRs) initially found to mediate the chemotaxis and activation of immune cells. During the past decades, the functions of these GPCRs have been discovered to not only regulate leukocyte trafficking and promote immune responses, but also play important roles in homeostasis, development, angiogenesis, and tumor progression. Accumulating evidence indicates that chemoattractant GPCRs and their ligands promote the progression of malignant tumors based on their capacity to orchestrate the infiltration of the tumor microenvironment by immune cells, endothelial cells, fibroblasts, and mesenchymal cells. This facilitates the interaction of tumor cells with host cells, tumor cells with tumor cells, and host cells with host cells to provide a basis for the expansion of established tumors and development of distant metastasis. In addition, many malignant tumors of the nonhematopoietic origin express multiple chemoattractant GPCRs that increase the invasiveness and metastasis of tumor cells. Therefore, GPCRs and their ligands constitute targets for the development of novel antitumor therapeutics.

## 1. Introduction

**Figure 1:**
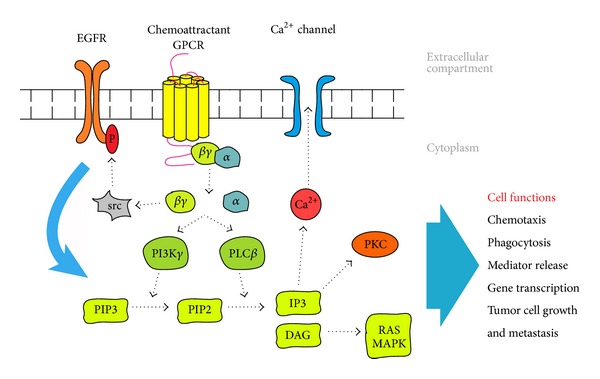
The signaling pathway of chemoattractant GPCRs. Chemoattractant GPCRs activated by ligands elicit a cascade of signal transduction pathways involving G proteins, phospholipase C (PLC), phosphoinositide (PI) 3 kinases, protein kinase C (PKC), Ca^2+^, RAS, and MAPKs to mediate leukocyte migration and activation. Chemoattractant GPCRs also play a crucial role in tumor progression upon activation by their ligands culminating in cell chemotaxis, invasion, production of mediators promoting angiogenesis, and transactivation of EGFR.

Chemoattractant receptors are a family of G protein coupled seven transmembrane cell surface receptors (GPCRs). According to their source of ligands and expression patterns, the family members are categorized into classical GPCRs and chemokine GPCRs. The former include formyl peptide receptor and its variants (FPR1, FPR2, and FPR3), platelet activating factor receptor (PAFR), activated complement component 5a receptor (C5aR), and leukotriene B4 receptor and its variants (BLT1 and BLT2). Chemokine GPCRs are composed of four subfamilies based on the conserved N-terminal cysteine residues in the mature proteins of the ligands, CC-, CXC-, CX3C-, and C-, and thus are termed CCR, CXCR, CX3CR, and XCR, respectively. So far, approximately 50 chemokines and at least 18 chemokine GPCRs have been identified [1] (Table 1). Promiscuity is a characteristic of GPCRs and their ligands. Some chemoattractants bind to more than one GPCR. Conversely, some GPCRs display overlapping ligand specificities with variable affinity and functions [2]. Although chemoattractant GPCRs are mainly expressed by leukocytes and their major function has been considered as mediators of leukocyte trafficking and homing, over the past two decades, the role of GPCRs and their ligands in tumor progression began to be increasingly recognized. The expression of some GPCRs or ligands in tumor tissues has been shown to be correlated with the therapeutic outcome of tumor patients [3–10]. It is undeniable that tumor cells are one of the major sources of chemoattractants in tumor tissues and many tumor cells express one or more chemoattractant GPCRs to their advantage [11]. In addition, tumor-derived chemoattractants are mediators of leukocyte, in particular macrophage (tumor-associated macrophages, TAMs), infiltration that may result in the persistence of chronic inflammation in the tumor microenvironment together with a vigorous angiogenesis. Therefore, chemoattractant GPCRs are believed to play a crucial role in tumor progression via signaling based on dissociation of trimeric G proteins in response to ligands binding culminating in cell chemotaxis, invasion, production of mediators promoting angiogenesis, transactivation of growth factor receptors, such as epidermal growth factor receptor (EGFR), and tumor cell metastasis. (Figure 1 shows the signaling.)

**Figure 2:**
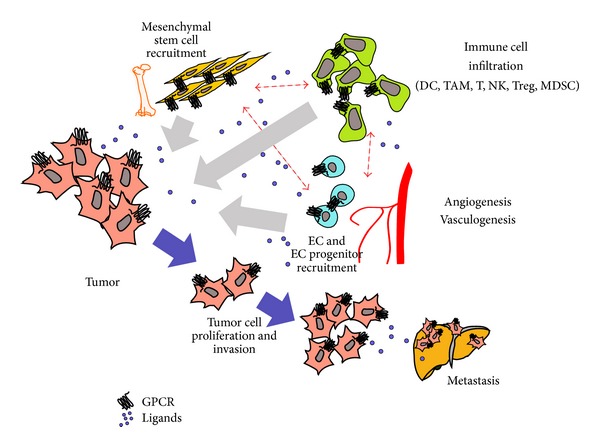
Chemoattractant GPCRs in tumor microenvironment. A tumor has been recognized as a complicated “organ.” Various types of tumor and host cells, including immune cells, fibroblasts, endothelial cells, and progenitor cells of the tumor stroma, contribute to the tumor development, growth, metastasis, immune escape, and neovascularization.

A tumor has been recognized as a complicated “organ,” other than a simple collection of relatively homogeneous cancer cells, whose entire biology could be understood by elucidating the autonomous properties of these cells. In contrast, various types of host cells are known to contribute in important ways to the biology of tumors, including endothelial cells (ECs), pericytes, immune cells, cancer-associated fibroblasts (CAFs), and stem and progenitor cells of the tumor stroma [54]. The interaction between these cells and their secreting factors results in an environment which markedly affects tumor progression. (Figure 2 shows the tumor.) Therefore, understanding the contribution of GPCRs and their ligands to the complexity of the tumor microenvironment is critical for the identification of novel therapeutic targets.

## 2. GPCRs in Recruiting Tumor-Associated Immune Cells

The infiltration of immune cells is a characteristic of the tumor microenvironment, which is the basis for the presence of chronic inflammation. Chemoattractants are characterized by their ability to induce directional migration and activation of leukocytes by stimulating specific GPCRs [2]. (Figure 1 shows the signaling.) The infiltrating immune cells play an important role in shaping a tumor-promoting or tumor-suppressive microenvironment [55, 56].

### 2.1. Tumor Infiltrating Tumor Suppressive Immune Cells

In general, infiltration of antigen presenting dendritic cells (DCs) into the tumor represents an early tumor-triggered host immune response. In hepatocellular carcinoma (HCC), tumor infiltrating DCs express the chemokine GPCRs, CCR1 and CCR5. Tumor cell apoptosis induced by suicide genes increases the number of DCs migrating into the draining lymph nodes to generate a specific cytotoxic cell population against HCC cells [57], although apoptotic tumor cells are also believed to generate tolerogenic DCs. In addition to CCR1 and CCR5, CCR6 is also commonly expressed by circulating immature DCs (iDCs). In melanoma, the profiling of GPCRs expressed by plasmacytoid DCs (pDCs) showed that the only significantly elevated GPCR is CCR6, which mediates the recruitment of pDCs from blood by the chemokine ligand CCL20 produced by melanoma cells [58]. Other immune cells in tumor microenvironment may also promote DC recruitment, such as Th9 cells, which increase DC infiltration of the tumor mediated by CCR6/CCL20 interaction that generates CD8(+) cytotoxic T lymphocyte (CTLs) responses and inhibit tumor growth [59]. After capturing antigens, DCs undergo maturation and express high levels of the chemokine GPCR CCR7 that enables DCs to migrate to T cell zones in the draining lymph nodes that produce the CCR7 ligands, CCL19 and CCL21. However, the results of interaction between DCs and tumor cells could be multifaceted based on CCR7/CCL19 or CCR7/CCL21 interaction [60, 61]. These chemokines may decide the distribution of immature or mature DCs within tumor tissues and generate opposing immunological consequences. For example, in renal cell carcinoma, tumor cells secrete CCL20 to recruit CCR6(+) immature DCs that mostly elicit tolerance, while, in the tumor invasion margin, only CCL19 and CCL21 are detected and they recruit CCR7(+) mature DCs as well as CCR7(+) T cells to form clusters that provide local foci of antitumor immune responses [62].

In addition to T cells, DCs may also cooperate with other immune competent cells, such as nature killer (NK) cells, to enhance antitumor effect. TLR9-activated pDCs could induce CTLs cross primed against multiple B16 tumor antigens, which is completely dependent on early recruitment and activation of NK cells. CCR5 expressing NK cells are recruited by CCL3, CCL4, and CCL5 secreted by pDCs, while IFN-*γ* was produced by NK cells stimulated by OX40L expressed on pDCs [63]. Conversely, IL-18-primed NK cells produce high levels of the iDC-attracting chemokines CCL3 and CCL4 to recruit iDCs in a CCR5-dependent manner and induce the production of CXCR3 and CCR5 ligands, CXCL9, CXCL10, and CCL5, by iDCs to facilitate the subsequent recruitment of CD8(+) T cells [64]. In breast cancer, NK cells take advantage of their own production of IFN-*γ* to enhance the secretion of chemokines CXCL9, CXCL10, and CXCL11 by tumor cells, which in turn accelerate the infiltration of CXCR3 expressing NK cells into the tumor site [65]. Hence, a positive feedback of DCs, NK cells, and tumor cells may result in the enhancement of antitumor immune responses. In addition, CCR5 and CXCR3 expressing CD8(+) T cells recruited by DCs are predominantly of the Th1 type that executes antitumor effect and colocalizes with macrophages and neutrophils to amplify the cell-mediated immune responses [56].

### 2.2. Tumor Infiltrating Immune Suppressive Cells

Immune suppressive cells recruited into tumor microenvironment subvert the host defense and create a microenvironment favoring tumor escape. These cells include myeloid-derived suppressor cells (MDSCs), TAMs, and regulatory CD4(+) T cells (Tregs). For example, in a melanoma model, when CTLs are injected intravenously into tumor-bearing mice, the cells are detected in the tumor as early as on day 1, peaking on day 3, and inhibit tumor growth. However, the antitumor effect is soon diminished with accumulation of MDSCs in the tumor, which outnumber CTLs by day 5. MDSCs produce nitric oxide, arginase I, and reactive oxygen species that inhibit the proliferation of antigen-specific CD8(+) T cells and reduce tumor cell killing. In CCR2−/− mice, the accumulation of MDSCs is significantly reduced, indicating that MDSC infiltration in the tumor is dependent on the chemokine GPCR CCR2 and its ligands, mainly CCL2 produced in the tumor [66].

Different T cell types appear in tumors at different stages of progression. In human ovarian cancer, recruitment of high numbers of Th1 cells was observed in stage II tumors, whereas activated Tregs along with high numbers of monocytes/macrophages and myeloid DCs (mDCs) were observed in disseminated tumors (stages III-IV). All tumor cells, monocytes/macrophages, and mDCs produce CCL22 to recruit Tregs via the GPCR CCR4. The specific recruitment of Tregs results in immune suppression in the advanced stages of ovarian cancer [67]. The paradox that early stage tumors are inhibited by infiltrating antitumor immune cells which is reversed by suppressive Tregs through CCR4/CCL22 interaction is also observed in myeloma [68]. Thus, chemokines and GPCRs play a crucial role in regulating pro- and antitumor responses by recruiting different types of immune cells (Table 2).

#### 2.2.1. Tregs

Treg is a CD4(+)CD25(+)FoxP3(+) T cell subtype. Treg expresses chemokine GPCR CCR4 and responds to the ligands CCL1 and CCL22 to accumulate in tumors. The degree of Treg infiltration is correlated with the prognosis of tumor patients [108, 110, 135]. A similar prognostic value was also obtained by the ratio of CD8(+) T cell/CCR4(+) Treg [136]. In melanoma, deletion of CD45RA(−)FoxP3(hi)CD4(+) Tregs (effector Tregs) using anti-CCR4 antibody significantly augmented CD8(+) T cell infiltration in the tumor and unmasked a nascent antitumor host response [137]. The recruitment of Tregs into the tumor microenvironment depended on the presence of CD8(+) T cells that produce ligands for CCR4 [138]. Therefore, the balance of infiltrating CCR4(+) Tregs and CD8(+) T cells in tumor tends to be a seesaw. Tregs can also interact with other cells in the tumor microenvironment. For instance, in a highly metastatic breast cancer model, only a proportion of CCR4(+) tumor cells in the primary tumor establish lung metastasis. Implanted orthotopic primary tumors “remotely” stimulate the expression of CCL17 and CCL22 in the lungs, which attract both CCR4(+) Tregs and tumor cells. CCR4(+) Tregs protect CCR4(+) tumor cells from being attacked by antitumor host immune cells. In fact, in the absence of CCR4(+) Tregs, CCR4(+) tumor cells disseminated into the lung are efficiently eliminated by NK cells, because CCR4(+) Tregs directly kill NK cells using beta-galactoside-binding protein [139]. Interestingly, in return, NK cells themselves also may attract Tregs through the CCR4/CCL22 interaction. In a Lewis lung cancer (LLC) implantation model, mouse lungs bearing LLC secrete CCL22 to recruit Tregs to suppress the proliferation of endogenous CD4(+)CD25(−) cells and the only cell type in the lung to produce CCL22 is NK cells [140]. CCR4/CCL22 even induces Tregs to selectively infiltrate into a particular site in the tumor, such as the area of lymphoid aggregates where Tregs are activated and proliferate in response to tumor-associated antigens presented by DCs. However, this process does not occur in the tumor bed [98, 141]. In addition, there are other GPCRs and ligands that may recruit Tregs, such as CCR5/CCL5 in colorectal cancer (CRC) and pancreatic cancer [104, 106], CCR6/CCL20 in HCC and breast cancer [108, 110], and CCR10/CCL28 in ovarian cancer [35], while CXCR3 and CXCR6 are expressed by Tregs infiltrating renal cell carcinoma [107]. Since Tregs are believed to be one of the major suppressive host cells that interfere with antitumor immune response, targeting GPCRs should be one of the effective measures to diminish Treg infiltration of the tumor environment thereby restoring tumor immunity.

#### 2.2.2. TAMs

In addition to the complicated interaction between Tregs and other tumor suppressing immune cells in the microenvironment, there are also other tumor supporting immune cells as important constituents. In a mouse CRC model, CCR6(+) Tregs are recruited into the tumor by responding to CCL20 secreted not only by tumor cells but also by TAMs. After targeted deletion of TAMs, Treg recruitment was abrogated with reduced tumor growth [109].

Macrophages are a major tumor infiltrating immune cell type that may affect tumor growth by either anti- or protumor effects [142]. Blood-derived monocytes infiltrate tumor tissues and differentiate into macrophages followed by further polarization into M1 or M2 phenotype, which differs in their patterns of cytokine secretion and biological function [143]. M1 macrophages mediate tumor suppression through type I cytokine production and tumor antigen acquisition and presentation [142, 144], whereas M2 macrophages promote tumor progression by producing type II cytokines [145]. Unfortunately, TAMs largely are of the M2 phenotype and promote the progression of almost all known solid tumors. Tumors produce many cytokines and other mediators that propel TAMs into the M2 phenotype [146]. Chemoattractant GPCRs are critical for TAM infiltration in the tumor, including chemokine GPCRs and the classical GPCR PAFR [84]. In certain tumor models, phagocytosis of apoptotic tumor cells by macrophages may induce M2 polarization, with the production of anti-inflammatory mediators [84, 147]. The main GPCR and ligand favoring TAM accumulation are CCR2/CCL2, which occurs in numerous tumors, such as pancreatic cancer, cervical cancer, papillary thyroid cancer, and prostate cancer [86, 91–93]. Some tumors also secrete other CCR2 ligands to recruit TAMs, such as HBD-3 in oral cancer [90]. In breast cancer, CCR2/CCL2 interaction recruits macrophages into the lung, where the cells “create” an appropriate microenvironment to facilitate tumor cell lodging and the development of metastatic foci [148]. FPR2 is also a GPCR expressed mainly on macrophages and neutrophils with the capacity to respond to bacterial chemotactic peptides [12]. In the mouse LLC model, tumors implanted subcutaneously grow more rapidly in mice deficient in Fpr2, the orthologue of human FPR2, and show significantly increased infiltration of TAMs with M2 polarization. Macrophages derived from Fpr2 deficient mice express higher levels of the chemokine GPCR, CCR4, which in cooperation with CCR2 mediate a marked increase in macrophage chemotaxis in response to CCL2. In addition, macrophages from Fpr2 deficient mice are more prone to M2 polarization after stimulation with LLC-derived supernatant. In contrast, in the presence of Fpr2, some macrophages develop an M1 phenotype after conditioning with LLC supernatant. Therefore, Fpr2 appears to sustain M1 differentiation of macrophages which participate in anti-LLC host responses [94]. Similarly, mice deficient in the chemokine GPCR CXCR3 exhibit polarization of TAMs into M2 phenotype in breast cancer [42]. Another chemokine GPCR, CX3CR1, and its ligand, CX3CL1, recruit TAMs and sustain the survival of TAMs to promote tumor metastasis [96, 149]. Therefore, chemoattractant GPCRs, in addition to mediating TAM recruitment, also favor TAM polarization to the M2 phenotype in response to tumor microenvironmental factors that promote tumor growth.

#### 2.2.3. MDSCs

Another type of immunosuppressive cells that shape the protumor microenvironment is MDSCs, which consist of subsets of immature myeloid cells with either monocytic or granulocytic morphology [150]. MDSCs are recruited into tumors via the chemokine GPCRs CCR2, CXCR2, or CXCR4 and are believed to promote tumor progression, such as facilitating metastasis in CRC [151, 152]. MDSCs exert their protumor activity by suppressing antitumor effectors, as by inhibiting T cell function via iNOS and arginase [80, 153, 154]. Deletion of CCR2(+) MDSCs using a toxin-mediated ablation strategy increased recruitment of activated CD8(+) T cells into the tumor and thus restored antitumor defense [150]. MDSCs are also capable of sustaining a protumor microenvironment by recruiting Tregs via chemoattractant GPCRs and ligands. For instance, MDSCs release CCL3, CCL4, and CCL5, which activate CCR5 expressed by Tregs and result in their recruitment in both in vitro and in vivo experimental models [105]. In addition to recruiting Tregs, a group of CD11b(+)CCR8(+) myeloid cells similar to MDSCs recruited by CCR8/CCL1 interaction in urothelial and renal carcinomas also “educate” tumor infiltrating T cells to express FoxP3, a marker for Tregs [31]. Thus, MDSCs have been recognized as an important component in the tumor microenvironment that are regulated by chemoattractant GPCRs and ligands. MDSCs also utilize the GPCR/ligand interactions to amplify protumor host response.

#### 2.2.4. Other Tumor Infiltrating Cells

In addition to immune cells, stromal cells in the tumor microenvironment also take part in the regulation of tumor growth. Mesenchymal stem cells (MSCs) are one of the major components in the tumor stroma and are believed to be the precursors of CAFs [155, 156]. MSCs may be recruited into the tumor through FPR2, CCR2, CXCR1, CXCR2, CXCR4, CXCR6, and CX3CR1 depending on the types and locations [125, 126, 128, 133]. Tumor-resident MSCs are often constantly exposed to immune cells and inflammatory cytokines in the microenvironment. They may have acquired functions distinct from normal tissue MSCs that alter the balance of host tumor interaction [88]. For example, compared with bone marrow MSCs, MSCs isolated from spontaneous mouse lymphomas (L-MSCs) promote tumor growth in association with recruitment of large numbers of CD11b(+) Ly6C(+) monocytes, F4/80(+) macrophages, and CD11b(+) Ly6G(+) neutrophils into the tumor. Depletion of monocytes/macrophages, but not neutrophils, completely abolishes the tumor promoting activity of L-MSCs. Such tumor infiltrating monocytes/macrophages are recruited by CCL2 produced by L-MSCs and CCR2 expressed on TAMs [88]. Similarly, CAFs are associated with immune suppressive microenvironment. In Hodgkin lymphoma and cutaneous T cell lymphoma, CAFs secrete the chemokines CCL11 and CCL26 that recruit CCR3(+) T lymphocytes into the tumor and produce high levels of IL-4, a signature of a Th2-dominant microenvironment [157].

In conclusion, GPCRs and ligands are critical for the recruitment of a variety of immune and nonimmune cells into the tumor microenvironment where these cells interact to establish host responses, which, unfortunately, mostly tip the balance to protumor elements.

## 3. The Role of Chemoattractant GPCRs Expressed by Tumor Cells

While chemoattractant GPCRs contribute to tumor growth by promoting the recruitment of protumor stromal cells and angiogenesis, many tumor cells also express a variety of GPCRs, which, by responding to autocrine and/or paracrine agonists produced in the microenvironment, directly stimulate tumor cell proliferation and tumor spread and expansion (Table 3).

In anaplastic large cell lymphomas, the CCR3/CCL11 interaction promotes tumor cell proliferation and inhibits apoptosis through ERK1/2, Bcl-xL and the production of survivin [192]. Similarly, through an AKT signaling pathway, CCR7 and its ligands CCL19 and CCL21 induce squamous cell carcinoma of the head and neck growth in vitro and in vivo [230]. In addition, CCR6/CCL20 interaction in endometrial adenocarcinoma, CXCR1/2/CXCL7 interaction in clear cell renal cell carcinoma, CXCR2/CXCL8 interaction in nasopharyngeal carcinoma, and CXCR6/CXCR16 interaction in HCC are reported to promote tumor cell growth [3, 27, 37, 265]. Hypoxia, which occurs during tumor expansion, induces the production of GPCR ligands that promote tumor cell proliferation in an autocrine manner. In cervical carcinoma, hypoxia stimulates tumor cells to express high levels of CXCR1/2 and CXCL8 that respond to ligands in the microenvironment by proliferating [246]. Actually, numerous chemoattractant GPCRs, such as CCR1, CCR5, CXCR5, CXCR7, and PAFR, are expressed by various types of tumor cells and are implicated in tumor growth [1]. In the case of the same GPCR, CXCR3, its two variants have opposite functions. CXCR3-A promotes cells growth but CXCR3-B mediates growth-inhibitory signals and induces apoptosis in various tumors [270].

In addition to tumor cells, stromal cells in the microenvironment also secrete GPCR ligands that stimulate the receptors on tumor cells in a paracrine manner which may represent a more important yet complicated stimulating loop. This is exemplified by observations in human glioma in which CXCR4/CXCL12 interaction favors an autocrine or paracrine loop for tumor cell proliferation [314, 315]. CXCR4/CXCL12 growth stimulating effects were also detected in glioma stem cells via an AKT-mediated prosurvival and self-renewal pathway. Highly malignant human glioblastoma cells (GBM) express a classical chemoattractant GPCR, FPR1, which recognizes a ligand, Annexin A1, released by necrotic GBM cells that mediates the proliferation of live GBM cells to increase their invasiveness and the production of angiogenic factors vascular endothelial growth factor (VEGF) and CXCL8 (IL-8), which stimulate VEGF receptor (VEGFR) and CXCR1/CXCR2 on vascular ECs to promote their migration and formation of new vasculature [316, 317]. It is interesting to note that FPR1 in GBM cells does not act alone; instead, the GPCR transactivates EGFR which accounts for part of the GBM growth stimulating activity of FPR1. GBM cells are able to maximally exploit the supportive mediators in the microenvironment to their advantage [1, 318]. By stimulating GPCR, tumor cells may even change the phenotype of neighboring stromal cells. Breast tumor cells secrete CCL20 to activate the ERK1/2/MAPK pathway in surrounding “normal” breast epithelial cells via CCR6 and promote their malignant transformation [319].

CAFs have been recognized as important regulators of tumor initiation by secreting CXCL12 to activate CXCR4 on breast cancer cells and stimulate tumor growth [320]. Studies have also shown that, after activation by CXCL12, breast cancer cells secrete another chemokine CCL20 that activates CCR6 expressed by tumor cells and facilitates their proliferation [321], while, in Hodgkin lymphoma, CAFs from tumor-involved lymph nodes cocultured with Reed-Sternberg cells produce CCL5, which activates CCR5 on tumor cells to stimulate tumor growth [205]. Multiple myeloma (MM) cells and osteoclasts (OCs) form yet another example of tumor promoting activity of GPCR/ligand interactions. MM growth in the bone marrow niche depends on bone resorption and interaction with active OCs [322, 323]. MM cells secrete CCL3 to activate OCs through its receptor CCR1 [324]. CCR1/CCL3 interaction inhibits the function of osteoblasts (OBs), resulting in the loss of OB/OC balance, which could facilitate MM growth [325]. Also, OCs in the tumor microenvironment sustain MM cell proliferation through production of chemokine that activate CCR2 on tumor cells [187]. These pathways culminate in MM outgrowth.

Based on these observations, it is now clear that chemokine GPCRs expressed by tumor cells and autocrine or paracrine ligands form a formidable interaction in the microenvironment that orchestrates the crisscross interaction between tumor cells and stromal cells stimulating further growth of the tumors.

## 4. The Role of Chemoattractant GPCRs in Tumor Metastasis

Metastasis is the major cause of cancer death. In order for cancer cells to metastasize, the cells should acquire a motile phenotype and be able to detach from the primary tumor mass to degrade basement membrane and intravasate into the blood or lymph vessels. After trafficking in the blood or lymphatic vessels, tumor cells tend to form emboli extravasating into distant organs or lymph nodes [1, 326]. Nearly each step of metastasis is heavily dependent on the tumor microenvironment and chemoattractant GPCRs are active participants in the processes.

A historical discovery of the role of chemoattractant GPCR/ligand interactions in promoting cancer metastasis was reported in 1998, in which the chemokine CCL2 (MCP-1) was shown to mediate kidney specific metastasis of a subpopulation of a murine experimental lymphoma [327]. This was followed by a more detailed study of several human cancer cell lines including breast and lung cancer cells which metastasized into distant organs in nude mice by using several chemokine GPCRs. These findings enriched the “seed” and “soil” paradigm of cancer metastasis by including chemoattractant GPCRs as the requisite for tumor cells as qualified “seeds” and a ligand producing distant organ or draining lymph nodes as suitable “soil” [328]. Since then, studies of the role of chemoattractant GPCRs and ligands in cancer metastasis have become a burgeoning research field and many malignant tumors have been shown to utilize a variety of GPCR/ligand interactions for metastasis. For example, in lung cancer, hypoxia induces the expression of CCR7 by tumor cells that increases cell invasiveness and eventual lymph node metastasis [29]. Hypoxia also promotes lymph node metastasis of breast cancer by increasing the expression of CCR5 on tumor cells and the ligand CCL5 in lymph nodes via the transcription factor hypoxia-inducible factor- (HIF-) 1*α* [25]. In prostate cancer and pancreatic ductal adenocarcinoma, cancer metastasis is associated with CX3CR1 on tumor cells and the ligand CX3CL1 at metastasis site [306, 308]. The sources of chemoattractants in tumor microenvironment are from both tumor and stromal cells. In prostate cancer, hypoxia-preconditioned MSCs produce CCL21 to attract tumor cells expressing CCR7 which is associated with enhanced lymph node metastasis of the tumor [229]. Similarly, under hypoxia, MSCs promote breast cancer metastasis through CXCR3/CXCL10 interaction [271].

Chemoattractant GPCRs and their ligands reportedly involved in enhanced tumor metastasis are listed in Table 4. Recently, cancer stem cells (CSCs) have been shown to account for most of the cancer metastasis. Interestingly, chemoattractant GPCRs participate in the maintenance of the metastatic property of CSCs by forming an autocrine loop. In ovarian cancer, the invasiveness of CD133(+) CSCs is enhanced by the chemokine CCL5, which activates CCR3 and CCR5 expressed by the cells to increase matrix metalloproteinase (MMP) 9 secretion [184]. A number of studies that use exogenous chemokines to induce cell invasion are in the literature. However, there are also a small number of chemokine and GPCR interactions that may inhibit tumor cells invasion, such as CX3CR1/CX3CL1 interaction in glioma [304].

While the aberrant expression of chemoattractant GPCRs is an important feature for a motile phenotype of tumor cells, the next step of tumor cell metastasis from the primary mass is detachment. These cells must survive the loss of interactions with extracellular matrix (ECM) that causes anoikis for further invasion of blood or lymph vessels [217]. In breast cancer, the activation of both CXCR4/CXCL12 and CCR7/CCL21 may reduce the sensitivity of metastatic cancer cells to anoikis by upregulating antiapoptotic proteins. Consequently, blocking the chemokine and GPCR interactions attenuates breast cancer metastasis in vivo [217]. Recently, another classical chemoattractant GPCR, BLT2, has also been shown to establish resistance to anoikis in prostate cancer cells through a BLT2-NOX-ROS-NF-*κ*B cascade [176].

Thus, accumulating evidence indicates an essential role of chemoattractant GPCRs and ligands in every step of cancer metastasis, including the acquisition of increased motility, detachment from the primary tumor mass by breaking down matrix proteins, intra- and extravasation, and lodgment in distant organs and lymph nodes. In addition, chemoattractant GPCRs and ligands also orchestrate the interaction of metastatic tumor cells with stromal cells, such as TAMs, ECs, and fibroblasts, which act either as “driving forces” for tumor cell dissemination or as “conditioners” of the “soil” that facilitates the settlement of metastatic tumor cells to develop secondary foci. Therefore, chemoattractant GPCRs and ligands provide promising molecular targets for prevention of tumor metastasis.

## 5. The Role of Chemoattractant GPCRs in Tumor Neovascularization

Neovascularization is critical for consolidation of the tumor microenvironment for tumor progression. Chemoattractant GPCRs provide pro- and antiangiogenic factors and receptors and are able to regulate two phases of neovascularization: vasculogenesis and angiogenesis (Table 5).

### 5.1. Vasculogenesis

Vasculogenesis is the formation of new blood vessels from circulating bone marrow-derived endothelial progenitor cells (EPCs). Coordinated events are required for the recruitment and incorporation of EPCs into the tumor tissue, including migration, invasion, differentiation, proliferation, and formation of vessels [461]. Although VEGF is a well-known angiogenic factor taking part in the vasculogenesis, other paracrine factors, such as chemoattractants produced by tumor cells, are also involved. EPCs expressing CXCR4 are mobilized by the ligand CXCL12 in an autocrine or paracrine manner [503]. Another chemokine CCL2 also mobilizes EPCs from the bone marrow [504]. These chemokines then promote EPC proliferation and guide the cells into tumor stroma to form functional neovasculature [505]. EPCs participating in neovascularization have also been reported in HCC, in which myeloid-derived EPCs (colony forming unit-endothelial cells) as early EPCs highly express CCR6 and are mobilized by the ligand CCL20 produced by HCC cells for migration and invasion of tumor stroma to form vasculature. CCR6/CCL20 in tumor microenvironments in addition plays a crucial role in driving phenotypic switch of hematopoietic cells with increased potential for angiogenic EC differentiation and attenuated proinflammatory activity [461]. A classical chemoattractant receptor, FPR1, may also participate in vasculogenesis in human GBM. This was shown in a xenograft model in which the number of EPCs incorporated into intracranial GBM lesion was significantly reduced in tumors formed by GBM cells in which FPR1 was depleted by RNA interference. The EPC chemotactic and tubule-stimulating activities were also attenuated in the supernatant of GBM cells deficient in FPR1 [160]. Another classical chemoattractant GPCR, the FPR1 variant FPR2, has also been reported to participate in recruiting MSCs into tumor tissues to promote the formation of neovasculature in response to tumor-derived ligand LL-37 [123].

### 5.2. Angiogenesis

Angiogenesis is a process in which new blood vessels sprout from existing vasculature. In tumor microenvironment, various cells regulate this process through GPCRs, which are expressed on vascular ECs and mediate cell recruitment and proliferation thereby extending the new vasculature in response to the ligands produced by tumor and other stromal cells. Tumor cells, tumor stem cells, and infiltrating TAMs in particular also express GPCRs capable of promoting the release of proangiogenic factors recruiting and activating vascular ECs [1].

FPR1 selectively expressed by GBM cells when activated by exogenous and tumor derived agonists promotes tumor cells to produce proangiogenic factors VEGF and the angiogenic chemokine CXCL8 [161, 316, 506]. CXCR1/2 expressed by vascular ECs and CXCL8, the ligand produced by tumor and stromal cells, are known to promote angiogenesis through inducing EC migration and formation of tubules [484, 507]. GBM stem cells may also utilize chemoattractant GPCRs FPR1 and CXCR4 to participate in angiogenesis by releasing VEGF [162, 249].

In addition to the direct interaction between chemoattractant GPCRs expressed by ECs and ligands in the tumor microenvironment, tumors take the advantage of infiltrating stromal cells, such as CAFs, TAMs, and Tregs, to benefit angiogenesis through GPCRs. In lung cancer, CAFs express CCR5 and are activated by CCL3 to secrete hepatocyte growth factor (HGF) to accelerate angiogenesis [326]. CAFs also cooperate with tumor cells to promote angiogenesis through CXCR4 expressed by both cell types. In pancreatic cancer, tumor cells secrete CXCL8 and CAFs secrete CXCL12 to enhance the recruitment and proliferation of ECs. However, CXCL12 promotes EC infiltration and CXCL8 enhances tubule formation by ECs revealing distinct functions of the CXCR2/CXCL8 and CXCR4/CXCL12 interactions in the process [269].

In addition, TAMs are an important source of angiogenic factors in tumor. For example, CCR2 and CD40 on TAMs are activated by CCL2 and CD40L produced in gastric cancer tissues and synergistically promote VEGF production to increase microvessel density [381, 508]. Moreover, Tregs expressing CCR10 are capable of accelerating angiogenesis through secreting VEGF in response to CCL28 produced by hypoxic tumor cells for EC infiltration and participation in angiogenesis [35].

It is interesting to note that alcohol consumption contributes to increased breast cancer angiogenesis, thus promoting the growth and metastasis of tumor cells in an animal model. This involves upregulated expression of CCR2 and CCL2 by tumor cells that increase the interaction between tumor and vascular ECs [467]. Another physical and chemical factor, radiation, exerts a similar effect through CXCR4/CXCL12 interaction on tumor angiogenesis [509].

Conversely, some chemoattractant GPCRs, such as CXCR3, are reported to mediate angiostatic activity through non-ELR CXC chemokines CXCR4/9/10/11 in various tumors [1]. The controversial results of angiogenesis are also found in C5aR [16, 17]. Therefore, angiogenesis may be regulated by a complex balancing process between opposing pro- and antiangiogenic GPCR and ligand interactions.

## 6. Perspectives

Accumulating evidence indicates crucial roles of chemoattractant GPCRs and their ligands in tumor progression by shaping tumor microenvironment. Almost all cell types including tumor cells per se are able to take the advantage of GPCRs and ligands to affect tumor progression. Chemoattractant GPCRs and ligands are involved in almost every step of tumor development and progression such as increasing tumor cell motility, invasiveness, intra- and extravasation, dissemination, leukocyte infiltration, and angiogenesis. These render the GPCRs and ligands promising drug targets for disruption of the tumor progression cascade. Recently, new agents targeting chemoattractant GPCRs have been developed and are being tested in the clinic, such as a humanized anti-CCR4 monoclonal antibody, mogamulizumab (KW-0761), aiming at curtailing cutaneous T cell lymphoma [510]. Therefore, gaining a better understanding of the GPCRs and their ligands in tumor microenvironment is vital and will provide novel therapeutic opportunities.

## Figures and Tables

**Table 1:** Chemoattractant GPCRs and ligands.

	Expression	Ligands	Functions	References
*“Classical” *				
FPR				
FPR1	Myeloid cells, lymphocytes Tumor cells	Bacteria and host derived peptides	Chemotaxis and activation Tumor growth, invasion, angiogenesis	[12, 13]
FPR2	Myeloid cells Tumor cells	Bacteria and host derived peptides	Chemotaxis and activation Antitumor defense, tumor invasion	[13]
FPR3	Monocytes, dendritic cells Tumor cells	Synthetic and host derived peptides	Chemotaxis and activation Tumor invasion	[13]
PAFR	Macrophages, polymorphonuclear leucocytes, and various tissue cells Tumor cells	PAF	Chemotaxis and activation Tumor growth and metastasis; inhibiting tumor angiogenesis	[14, 15]
C5aR	Neutrophils, monocytes, eosinophils, basophils, dendritic cells, mast cells, and various nonimmune cells Tumor cells	C5a	Chemotaxis and activation Tumor metastasis; opposite function in angiogenesis	[16–18]
LTB4R				
BLT1	Neutrophils, macrophages, T lymphocytes Tumor cells	LTB4	Chemotaxis and activation Tumor growth	[19]
BLT2	Most human tissues cells and leukocytes Tumor cells	LTB4	Chemotaxis and activation Tumor growth, metastasis	[19]
*“Chemokine” *				
CCR				
CCR1	Monocytes, neutrophils, T lymphocytes, dendritic cells Tumor cells	CCL3/4/6/7/8/9/10/14/15/16/23	Chemotaxis and activation Tumor growth, metastasis, angiogenesis	[20]
CCR2	Monocytes, basophils, T lymphocytes, dendritic cells, NK cells, endothelial cells Tumor cells	CCL2/7/8/11/13/16	Chemotaxis and activation Tumor growth, metastasis, angiogenesis	[21, 22]
CCR3	Eosinophils, basophils, Th2 lymphocytes, mast cells Tumor cells	CCL7/11/13/15/24/26/28	Chemotaxis and activation Tumor growth, metastasis	[23]
CCR4	Macrophages, monocytes, basophils, T and B lymphocytes, dendritic cells, NK cells, mast cells, platelets Tumor cells	CCL2/4/5/17/22	Chemotaxis and activation Tumor growth, metastasis, angiogenesis	[24]
CCR5	Macrophages, T lymphocytes, dendritic cells, NK cells Tumor cells	CCL3/4/5/7/11/13/16	Chemotaxis and activation Tumor growth, metastasis, angiogenesis	[25, 26]
CCR6	Neutrophils, T and B lymphocytes, dendritic cells, epithelial cells of some tissues Tumor cells	CCL20	Chemotaxis and activation Tumor growth, metastasis	[27, 28]
CCR7	T and B lymphocytes, dendritic cells Tumor cells	CCL19/21	Lymphoid tissue chemotaxis and activation Tumor growth, metastasis	[29, 30]
CCR8	Macrophages, Th2 lymphocytes, endothelial cells Tumor cells	CCL1/16	Chemotaxis and activation Tumor metastasis	[31, 32]
CCR9	T lymphocytes Tumor cells	CCL25	Small intestinal specific chemotaxis and activation Tumor growth, metastasis; inhibiting tumor metastasis in some tumors	[33, 34]
CCR10	T lymphocytes Tumor cells	CCL27/28	Skin-specific chemotaxis and activation Tumor growth, metastasis, angiogenesis	[35, 36]
CXCR				
CXCR1	Neutrophils, polymorphonuclear leukocytes, endothelial cells Tumor cells	CXCL6/8	Chemotaxis and activation Tumor growth, metastasis, angiogenesis	[37–39]
CXCR2	Neutrophils, basophils, T lymphocytes, oligodendrocytes, endothelial cells Tumor cells	CXCL1/2/3/5/6/8	Chemotaxis and activation Tumor growth, metastasis, angiogenesis	[40, 41]
CXCR3	Macrophages, T lymphocytes, NK cells, NKT cells, endothelial cells Tumor cells	CXCL4/9/10/11	Chemotaxis and activation Two variants CXCR3-A and CXCR3-B have opposite function in tumor progression	[42, 43]
CXCR4	Numerous cell types: hematopoietic cells and stem cells Tumor cells	CXCL12	Chemotaxis and activation Maintenance of stem phenotype Tumor growth, metastasis, angiogenesis	[1, 44]
CXCR5	T and B lymphocytes Tumor cells	CXCL13	Chemotaxis and activation Tumor growth, metastasis; inhibiting tumor metastasis in some tumors	[45, 46]
CXCR6	T and B lymphocytes, NK cells, NKT cells, plasma cells Tumor cells	CXCL16	Chemotaxis and activation Tumor growth, metastasis, angiogenesis; inhibiting tumor migration in some tumors	[47]
CXCR7	T and B lymphocytes, dendritic cells, endothelial cells, fetal hepatocytes Tumor cells	CXCL11/12	Chemotaxis and activation Tumor growth, metastasis, angiogenesis; assisting with CXCR4 to regulate tumor progression	[48–50]
CX3CR				
CX3CR1	Monocytes, T and B lymphocytes, mast cells, dendritic cells, NK cells Tumor cells	CX3CL1	Chemotaxis and activation Tumor growth, metastasis; inhibiting tumor invasion in some tumors	[51, 52]
XCR				
XCR1	Neutrophils, T lymphocytes, dendritic cells Tumor cells	XCL1/2	Chemotaxis and activation Tumor cell growth, metastasis	[53]

**Table 2:** Chemoattractant GPCRs associated with stromal cell infiltration.

	GPCRs	Tumor types
Immune cells		

Dendritic cells	CCR1	Hepatocellular carcinoma [57]
	CCR5	Hepatocellular carcinoma [57], ovarian cancer [69]
	CCR6	Breast cancer [70, 71], colorectal cancer and lung cancer [72], lymphoma [73], melanoma [58, 72], lymphocyte-rich gastric cancer [74], renal cell carcinoma [62], thyroid cancer [75]
	CCR7	Breast cancer [76], renal cell carcinoma [62]
	CXCR1/2	Colorectal cancer [77–79], gastric cancer [79], hepatocellular carcinoma [78], pancreatic cancer [78]

Myeloid-derived suppressor cells	CCR2	Basal cell carcinomas [80], melanoma [66]
	CXCR2	Colitis-associated cancer [81]
	CXCR4	Gastric cancer [82], ovarian cancer [83]

Tumor-associated macrophages	PAFR	Melanoma [84]
	CCR2	Breast cancer [85], cervical cancer [86], colitis-associated cancer [87], lymphoma [88], nasopharyngeal carcinoma [89], oral cancer [90], prostate cancer [91], pancreatic cancer [92], thyroid cancer [93]
	CCR4	Lung cancer [94]
	CCR5	Hepatocellular carcinoma [95], nasopharyngeal carcinoma [89]
	CXCR3	Breast cancer [42]
	CX3CR1	Breast cancer [96], glioma [97]

Regulatory T cells	CCR4	Breast cancer [98], cervical cancer [99], Hodgkin lymphoma [100], gastric cancer [101], glioma [102], melanoma [103]
	CCR5	Colorectal cancer [104], lymphoma [105], pancreatic cancer [106], renal cell carcinoma [107]
	CCR6	Breast cancer [108], colorectal cancer [109], hepatocellular carcinoma [110], Hodgkin lymphoma [111], renal cell carcinoma [107]
	CCR7	Melanoma [112], ovarian cancer [113]
	CCR10	Ovarian cancer [35]
	CXCR1	Lung cancer, mesothelioma, melanoma [114]
	CXCR3	Renal cell carcinoma [107]
	CXCR4	Breast cancer [115], B cell lymphoma [116], hepatocellular carcinoma [117], lung cancer [118], glioma [119], ovarian cancer [120, 121]
	CXCR6	Nasopharyngeal carcinoma [122], renal cell carcinoma [107]

Stromal cells		

Mesenchymal stem cells	FPR2	Ovarian tumor [123]
	CCR2	Breast cancer [124], glioma [125], lymphoma [88]
	CXCR1	Glioma [126, 127]
	CXCR2	Kidney cancer [128], glioma [127]
	CXCR4	Breast cancer [129], gastric cancer [130], glioma [125, 131, 132]
	CXCR6	Glioma [132], prostate cancer [133]
	CX3CR1	Colorectal cancer [134]

**Table 3:** The functions of chemoattractant GPCRs expressed by tumor cells.

GPCRs	Tumor	Function	References
FPR			
FPR1	Colorectal cancer	Invasion	[158]
	Gastric cancer	Invasion	[159]
	Glioblastoma	Growth, invasion, vasculogenesis, angiogenesis	[160–163]
FPR2	Gastric cancer	Invasion	[159]
	Ovarian cancer	Invasion	[164]
FPR3	Gastric cancer	Invasion	[159]
PAFR	Breast cancer	Migration, proliferation, angiogenesis	[165]
	Melanoma	Metastasis	[166]
	Ovarian cancer	Proliferation, invasion	[167]
C5aR	Bile duct cancer, colorectal cancer	Invasion	[168]
	Non-small-cell lung cancer	Metastasis	[169]
LTB4R			
BLT1	Colorectal cancer	Proliferation	[170]
BLT2	Bladder cancer	Metastasis, antiapoptosis	[171, 172]
	Breast cancer	Metastasis	[173]
	Pancreatic cancer	Growth, migration	[174, 175]
	Prostate cancer	Antianoikis, antiapoptosis	[176, 177]
	Ovarian cancer	Metastasis	[178]
CCR			
CCR1	Breast cancer	Invasion	[179]
	Glioma	Proliferation, tumorigenesis	[180]
	Hepatocellular carcinoma	Migration, invasion	[181, 182]
	Oral squamous cell carcinoma	Migration	[183]
	Ovarian cancer	Invasion	[184]
CCR2	Bladder cancer	Migration, invasion	[185]
	Breast cancer	Migration, proliferation, antiapoptosis	[186]
	Hepatocellular carcinoma	Migration, invasion	[181]
	Multiple myeloma	Growth	[187]
	Ovarian cancer	Invasion, adhesion, proliferation	[188, 189]
	Prostate cancer	Proliferation, migration, invasion	[190, 191]
CCR3	Lymphoma	Growth	[192]
	Glioma	Proliferation, tumorigenesis	[180]
	Oral squamous cell carcinoma	Migration, invasion	[183]
	Ovarian cancer	Invasion, proliferation	[184, 189]
	Renal cell carcinoma	Growth, dissemination	[193]
CCR4	Breast cancer	Growth, metastasis, angiogenesis	[139, 194]
	Colorectal cancer	Migration	[195]
	Gastric cancer	Migration	[196]
	Melanoma	Metastasis	[197]
	Squamous cell carcinoma of the head and neck	Metastasis	[198]
CCR5	Breast cancer	Proliferation, metastasis	[25, 199–202]
	Colorectal cancer	Growth	[203]
	Gastric cancer	Metastasis	[204]
	Glioma	Proliferation, tumorigenesis	[180]
	Hodgkin lymphoma	Growth, metastasis	[205]
	Oral cancer	Migration	[206]
	Ovarian cancer	Invasion, proliferation	[189]
CCR6	Colorectal cancer	Proliferation, metastasis	[207, 208]
	Endometrial adenocarcinoma	Proliferation	[27]
	Hepatocellular carcinoma	Metastasis	[209, 210]
	Non-small-cell lung cancer	Metastasis	[211]
	Pancreatic cancer	Invasion	[212–214]
	Squamous cell carcinoma of the head and neck	Metastasis	[215, 216]
CCR7	Breast cancer	Metastasis, antianoikis	[217, 218]
	Colorectal cancer	Metastasis	[219, 220]
	Melanoma	Growth, metastasis, tumorigenesis	[221, 222]
	Non-small-cell lung cancer	Proliferation, antiapoptosis, metastasis	[29, 223–226]
	Oral squamous cell carcinoma	Metastasis	[227]
	Pancreatic ductal adenocarcinoma	Metastasis	[228]
	Prostate cancer	Metastasis	[229]
	Squamous cell carcinoma of the head and neck	Proliferation, antiapoptosis, metastasis, adhesion	[230–236]
	T cell lymphoma	Dissemination	[237]
CCR8	Melanoma, breast cancer, leukemia	Metastasis	[32]
CCR9	Breast cancer	Migration, invasion	[238]
	Colorectal cancer	Inhibiting metastasis	[239]
	Ovarian cancer	Migration, invasion	[240]
	Pancreatic cancer	Proliferation, invasion	[34, 241]
	Prostate cancer	Antiapoptosis	[242]
CCR10	Melanoma	Growth, metastasis	[243, 244]
CXCR			
CXCR1	Breast cancer	Stem cell self-renewal	[245]
	Cervical carcinoma	Proliferation	[246]
	Colorectal cancer	Metastasis, antiapoptosis, angiogenesis	[247]
	Gastric cancer	Invasion	[248]
	Glioblastoma	Growth, migration, invasion	[249, 250]
	Melanoma	Growth, migration, invasion, angiogenesis, tumorigenesis	[251–253]
	Prostate cancer	Growth, angiogenesis	[254]
	Renal cell carcinoma	Growth, angiogenesis	[37]
	Thyroid carcinoma	Metastasis	[255]
CXCR2	Breast cancer	Migration, invasion, stem cell self-renewal	[245, 256, 257]
	Cervical carcinoma	Proliferation	[246]
	Colorectal cancer	Proliferation, migration, invasion, angiogenesis	[258–261]
	Gastric cancer	Metastasis	[262, 263]
	Glioblastoma	Growth, migration	[249, 264]
	Melanoma	Growth, migration, invasion, angiogenesis, tumorigenesis	[251–253]
	Nasopharyngeal carcinoma	Growth	[265]
	Non-small-cell lung cancer	Growth, metastasis, angiogenesis	[266, 267]
	Ovarian cancer	Growth, angiogenesis	[268]
	Pancreatic cancer	Invasion, angiogenesis	[269]
	Prostate cancer	Growth, angiogenesis	[254]
	Renal cell carcinoma	Growth, angiogenesis	[37]
	Thyroid carcinoma	Metastasis	[255]
CXCR3	Breast cancer	Metastasis; inhibiting growth	[270–273]
	Colorectal cancer	Metastasis	[274]
	Glioma	Growth	[275, 276]
	Lung adenocarcinoma	Metastasis	[226]
	Melanoma	Migration	[277]
	Myeloma	Inhibiting/promoting proliferation and apoptosis	[43]
	Ovarian cancer	Growth, metastasis	[278]
	Prostate cancer	Metastasis	[279]
	Renal cell carcinoma	Growth, metastasis	[280, 281]
CXCR4	At least 23 haematopoietic and solid cancers	Growth, metastasis, angiogenesis	[1, 44]
CXCR5	Breast cancer	Metastasis	[282]
	Colorectal cancer	Growth, migration	[283]
	Neuroblastoma	Inhibiting/promoting metastasis	[45, 284]
	Prostate cancer	Proliferation, invasion, migration, adhesion	[285–288]
CXCR6	Colorectal cancer	Growth, migration, invasion	[289]
	Hepatocellular carcinoma	Growth, metastases, angiogenesis	[3]
	Melanoma	Stem cell self-renewal	[290]
	Nasopharyngeal carcinoma	Metastasis	[291]
	Pancreatic ductal adenocarcinoma	Invasion	[292]
	Prostate cancer	Proliferation, metastasis	[293–295]
	Renal cell carcinoma	Inhibiting migration	[296]
CXCR7	Breast cancer	Inhibiting invasion; growth, angiogenesis	[297]
	Cervical carcinoma	Growth, adhesion	[298]
	Glioma	Growth, migration, sphere and tube formation	[49, 299]
	Hepatocellular carcinoma	Growth, metastasis, angiogenesis	[300, 301]
	Lymphoma	Growth, adhesion	[298]
	Nasopharyngeal carcinoma	Metastasis	[291]
	Neuroblastoma	Inhibiting growth; metastasis	[50, 302]
CX3CR			
CX3CR1	Epithelial ovarian carcinoma	Proliferation, migration, adhesion	[303]
	Glioma	Inhibiting invasion	[304]
	Neuroblastoma	Migration	[305]
	Pancreatic ductal adenocarcinoma	Migration	[306, 307]
	Prostate cancer	Metastasis	[308–310]
	Renal cell carcinoma	Metastasis	[311]
XCR			
XCR1	Epithelial ovarian carcinoma	Proliferation, metastasis	[312]
	Oral squamous cell carcinoma	Proliferation, migration, invasion	[313]

**Table 4:** Chemoattractant GPCRs associated with tumor metastasis.

Tumor type	GPCRs	Ligands	Metastatic sites
Bladder cancer	BLT2	LTB4	Lung [171]
	CCR2	CCL2	Lung [329]
	CXCR6	CXCL16	Perineural and lymphovascular invasion [330]

Breast cancer	BLT2	LTB4	Lung [173]
	CCR2	CCL2	Lung [85, 148], bone [148]
	CCR4	CCL17/22	Lung [139, 194, 331]
	CCR5	CCL5	Lung [200, 201], lymph node [25, 332]
	CCR6		Pleura [333]
	CCR7	CCL19/21	Lymph node [218, 334–337]
			Skin [333]
	CCR8	CCL1	Lymph node [32]
	CCR9	CCL25	Lymph nodes and gastrointestinal tract [238]
	CXCR1	CXCL8	Bone [338, 339]
	CXCR2		Lung [340], bone [341]
	CXCR3	CXCL9	Lung [342]
		CXCL10	Bone [343], lung [344]
	CXCR4	CXCL12	Lymph node [328, 336, 337, 345], bone [346–348], lung [328, 346, 349], liver [333]
	CXCR5	CXCL13	Lymph node [282]
	CXCR6	CXCL16	Lymph node [350]
	CXCR7	CXCL12	Lung, greater omentum, and lymph nodes [351]
	CX3CR1		Brain [333]

Cervical cancer	CXCR4	CXCL12	Lymph node [352]
	CXCR4/7	CXCL12	Lymph node [353, 354]

Colorectal cancer	CCR1	CCL7/9/15	Liver [355–357]
	CCR2	CCL2	Liver [151, 358], lung [359]
		CCL7	Liver [356]
	CCR3	CCL7	Liver [356]
	CCR5	CCL5	Liver and lung [203]
	CCR6	CCL20	Liver [207, 360]
	CCR7	CCL21	Lymph node [219, 220, 361]
	CXCR1/2		Liver [247]
	CXCR2	CXCL1	Lymph node [261], liver [362]
		CXCL8	Skin [363]
	CXCR3	CXCL9	Lymph node [364]
		CXCL10	Lymph node [364], lung [365]
		CXCL11	Lung [365]
	CXCR6	CXCL16	Liver [289, 366]
	CXCR4	CXCL12	Liver [367–370], lymph node [371, 372], brain [373]

Esophageal cancer	CCR7	CCL21	Lymph node [374–376]
	CXCR2		Lymph node [377]
	CXCR4	CXCL12	Lung [378, 379], liver [378, 379], lymph node [378, 380], peritoneum [379], retroperitoneum [379]

Gastric cancer	FPR1/2/3	Annexin A1	Peritoneum [159]
	CCR2	CCL2	Lymph node [381]
	CCR4	CCL17	Lymph node, lung, and bone [194]
	CCR5	CCL5	Lymph node [204]
	CCR7		Lymph node [30, 382, 383]
	CXCR2	CXCL1	Lymph node [262]
	CXCR4	CXCL12	Lymph node [382, 384–387], peritoneum [388–390], liver [387]

Glioma	CXCR4/7	CXCL12	Bone marrow [299] Lymph node, distant organs [391]

Head and neck squamous cell carcinoma	CCR4	CCL22	Lymph node [198]
	CCR6	CCL20	Lymph node [216, 392]
	CCR7	CCL19/21	Lymph node [227, 230–232, 393]
	CXCR2	CXCL1/8	Lymph node [394, 395]
	CXCR4	CXCL12	Lymph node [393, 396], lung [397, 398]
	CXCR5	CXCL13	Bone [399]
	XCR1	XCL1	Lymph node [313]

Hepatocellular carcinoma	CCR7		Intrahepatic metastasis, lymph node [400]
	CXCR4	CXCR12	Lung [401], bone [402, 403], lymph node [404]
	CXCR6	CXCL16	Lung [3]
	CXCR7	CXCL12	Lung [300, 405]

Lymphoma	CCR7	CCL21	Lymph node [237]

Leukemia	CCR8	CCL1	Lymph node [32]
	CXCR4	CXCL12	Extramedullary sites (liver, kidney, spleens, and peripheral blood) [406]

Melanoma	FPR1/2/3	Annexin A1	Lung [407]
	PAFR	PAF	Lung [166, 408, 409]
	CCR2	CCL2	Lung [410]
	CCR3		Brain [411]
	CCR4	CCL22	Brain [197, 411]
	CCR5	CCL4	Lung [412, 413]
	CCR7	CCL21	Lymph node [221, 222, 244, 414], liver [415]
	CCR8	CCL1	Lymph node [32]
	CCR9	CCL25	Small intestinal [416, 417]
	CCR10	CCL27	Skin [243, 244]
	CXCR2	CXCL8	Lung [418]
	CXCR3	CXCL10	Lymph node [419, 420], bone [421]
	CXCR4	CXCL2	Lung [244, 422–424]

Neuroblastoma	CXCR3	CXCL10	Bone marrow [425]
	CXCR4	CXCL12	Bone [426–428], liver [429, 430], kidney [430], bone marrow [428, 430]
	CXCR5	CXCL13	Bone marrow [284]
	CXCR4/7	CXCL12	Bone marrow [302]
	CX3CR1	CX3L1	Bone marrow [305]

Non-small-cell lung cancer	C5aR		Lymph node [169]
	CCR4	CCL22	Bone [431]
	CCR6	CCL20	Adrenal specific metastasis [211]
	CCR7	CCL19/21	Lymph node [29, 226, 432]
	CXCR2	CXCL5	Hilar and mediastinal lymph nodes, chest wall, and contralateral lung; extrathoracic distant metastases (para-aortic lymph nodes, liver, adrenal glands, kidneys, spleen, and diaphragm) [266]
	CXCR4	CXCL12	Lungs, liver, bone marrow, adrenal glands [433], pleural [434], brain [433, 435]
	CX3CR1		Brain and liver [436]

Osteosarcoma	CCR7	CCL21	Lymph node [334]
	CXCR3	CCL9/10/11	Lung [437]
	CXCR4	CXCL12	Lung [438]
	CXCR7	CXCL12	Lung [439]

Ovarian carcinoma	BLT2		Diaphragm, intestine, and mesentery (intraperitoneal dissemination) [178]
	CCR3	CCL5	Liver, bowel, and spleen [184]
	CCR9	CCL25	Small intestinal [440]
	CXCR4	CXCL12	Pelvic [441], lymph node [442, 443], peritoneum [444]
	CXCR6	CXCL16	Lymph node [443]
	XCR1	XCL1/2	Diaphragm, peritoneal wall, colon, spleen, and liver [312], peritoneum [312]

Pancreatic cancer	CCR2	CCL2	Liver [92, 445], peritoneal [445]
	CCR7	CCL21	Lymph node [228, 446]
	CXCR4/7	CXCL12	Liver [447, 448], lung [448], lymph node [449]

Prostate cancer	CCR2	CCL2	Bone [450]
	CCR7	CCL21	Lymph node [229]
	CXCR1/2	CXCL8	Lymph node [451]
	CXCR3	CXCL4/10	Lymph node, liver, lung, adrenal [279]
	CXCR4	CXCL12	Bone [133, 452, 453]
	CXCR5	CXCL13	Bone [288]
	CXCR6	CXCL16	Bone [133, 294, 453], liver [294]
	CX3CR1	CX3CL1	Bone [310]

Renal cell carcinoma	CCR1/3	CCL15	Bone [454]
	CCR5	CCL3	Lung [326]

Thyroid papillary cancer	CCR7	CCL21	Lymph node [455, 456]
	CXCR1/2	CXCL8	Lymph node [255]
	CXCR4		Lymph node [455, 457, 458]
	CXCR7		Lymph node [459]

**Table 5:** Chemoattractant GPCRs associated with tumor neovascularization.

	Receptors	Tumors
Vasculogenesis	FPR1	Glioma [160]
	FPR2	Ovarian cancer [123]
	CCR2	Hepatocellular carcinoma [460]
	CCR5	Hepatocellular carcinoma [460]
	CCR6	Hepatocellular carcinoma [461]
	CXCR2	Pancreatic cancer [462]
	CXCR4	Breast cancer [320], melanoma [463]

Angiogenesis	FPR1	Glioma [161, 162, 316]
	C5aR	Epithelial ovarian cancer [17]
	CCR1	Hepatocellular carcinoma [464], lymphoma [465], multiple myeloma [466]
	CCR2	Breast cancer [22, 467, 468], esophageal cancer [469], gastric cancer [381], melanoma [470]
	CCR4	Breast cancer [194]
	CCR5	Multiple myeloma [466], renal cell carcinoma [326]
	CCR10	Ovarian cancer [35]
	CXCR1	Prostate cancer [471], renal cell carcinoma [37]
	CXCR2	Cervical cancer [472], colorectal cancer [258, 259], glioblastoma [473], lung adenocarcinoma [267, 474, 475], melanoma [418, 476], ovarian cancer [268], pancreatic cancer [269, 477–479], prostate cancer [480], renal cell carcinoma [37, 481]
	CXCR1/2	Glioblastoma [482], melanoma [251, 253], multiple myeloma [483], ovarian cancer [484], pancreatic cancer [485], prostate cancer [254, 451, 486], renal cell carcinoma [37]
	CXCR4	Breast cancer [487], colorectal cancer [488, 489], gastric cancer [490], glioblastoma [491–493], hepatocellular carcinoma [494], ovarian cancer [495], pancreatic cancer [269, 496], prostate cancer [497], squamous cell carcinoma [398]
	CXCR6	Hepatocellular carcinoma [3], prostate cancer [295]
	CXCR7	Bladder cancer [498], breast cancer [297], breast and lung cancer [499], colorectal cancer [488], hepatocellular carcinoma [301], prostate cancer [500], renal cell carcinoma [501]
	CX3CR1	Breast cancer [96], colorectal cancer [149], melanoma [502]
